# Hsa-miR-194-5p and hsa-miR-195-5p are down-regulated expressed in high dysplasia HPV-positive Pap smear samples compared to normal cytology HPV-positive Pap smear samples

**DOI:** 10.1186/s12879-023-08942-1

**Published:** 2024-02-12

**Authors:** Ali Dehghani, Fardin Khajepour, Mohammad Dehghani, Ehsan Razmara, Mohammadreza Zangouey, Maryam Fekri Soofi Abadi, Reza Bahram Abadi Nezhad, Shahriar Dabiri, Masoud Garshasbi

**Affiliations:** 1 Department of Medical Genetics, Faculty of Medical Sciences, Tarbiat Modares University, Tehran, Iran; 2 Department of Immunology, Afzalipour Faculty of Medicine, Kerman University of Medical Sciences, Kerman, Iran; 3 Pathology and Stem Cell Research Center, Kerman University of Medical Sciences, Kerman, Iran; 4 Australian Regenerative Medicine Institute, Monash University, Melbourne, Australia; 5 Department of Microbiology, School of Medicine, Rafsanjan University of Medical Sciences, Rafsanjan, Iran

**Keywords:** Cervical cancer, Cervical dysplasia, Human papillomavirus, Hsa-miR-194-5p, Hsa-miR-195-5p

## Abstract

**Background:**

The human papillomavirus (HPV) infection may affect the miRNA expression pattern during cervical cancer (CC) development. To demonstrate the association between high-risk HPVs and the development of cervix dysplasia, we examined the expression patterns of hsa-miR-194-5p and hsa-miR-195-5p in Pap smear samples from southeast Iranian women. We compared samples that were HPV-positive but showed no abnormality in the cytological examination to samples that were HPV-positive and had severe dysplasia.

**Methods:**

Pap smear samples were obtained from 60 HPV-positive (HPV-16/18) patients with histologically confirmed severe dysplasia (cervical intra-epithelial neoplasia (CIN 3) or carcinoma in situ) and the normal cytology group. The expression of hsa-miR-194-5p and hsa-miR-195-5p was analyzed by real-time quantitative PCR, using specific stem-loop primers and U6 snRNA as the internal reference gene. Clinicopathological features were associated with miRNA expression levels. Furthermore, functional enrichment analysis was conducted using in silico tools. The Kaplan–Meier survival method was also obtained to discriminate survival-significant candidate miRNAs in CC, and receiver operating characteristic (ROC) curves were constructed to assess the diagnostic value.

**Results:**

Compared to HPV-positive cytologically normal Pap smear samples, hsa-miR-194-5p and hsa-miR-195-5p relative expression decreased significantly in HPV-positive patients with a severe dysplasia Pap smear. Kaplan–Meier analysis indicated a significant association between the miR-194 decrease and poor CC survival. In essence, ROC curve analysis showed that miR-194-5p and miR-195-5p could serve as valuable markers for the development of cervix dysplasia in individuals who are positive for high-risk HPVs.

**Conclusions:**

This study revealed that hsa-miR-194-5p and hsa-miR-195-5p may possess tumor suppressor capabilities in the context of cervical dysplasia progression. However, it remains uncertain whether these microRNAs are implicated in the transition of patients with high dysplasia to cervical cancer. We also showed the potential capability of candidate miRNAs as novel diagnostic biomarkers related to cervical dysplasia progression.

## Introduction

Cervical cancer (CC) is a leading cause of mortality in women worldwide and represents a substantial global health challenge. In 2020, more than 604,000 new cases were detected worldwide, and about 342,000 women died, primarily middle-aged [1].

CC typically does not cause any symptoms in its early stages. However, as the cancer advances, it may lead to several noticeable symptoms, such as irregular vaginal bleeding, pelvic pain, and changes in the color or odor of vaginal discharge. These symptoms typically coincide with abnormal changes in the microscopic appearance of the cells on the surface of the cervix [2]. Although the exact etiology of CC is unknown, infection with the human papillomavirus (HPV), smoking, impaired immunity, and other risk factors have been linked to this disease [3]. CC is a multifactorial condition attributed to environmental, genetic, and epigenetic factors [4].

The conventional Papanicolaou (Pap) smear test has been the gold standard in screening cervical pre-malignant and malignant conditions since the 1950s. However, due to low sensitivity and considerable false-negative rate of cytological examinations, the US Preventive Services Task Force recommended a combination of cytology and molecular HPV evaluation (co-testing) [5]. Women worldwide continue to suffer from CC despite significant advances in screening methods in recent years [6]. Thus, a better understanding of the processes behind CC progression is critical to finding a conscious and solid method to improve CC diagnosis. Also, CC can be more effectively treated if precancerous lesions are found in early stages via cytological screening and CC-related molecular tests [7].

In numerous human cases, high-risk (HR) HPV infections [including HPV16 and HPV18] have been identified as substantial risk factors for CC development [8]. Long-term misexpression of HR HPV E6 and E7 oncogenes contributes to CC development [9]. These oncogenes trigger neoplastic cell transformation primarily because of genetic and epigenetic instability. On top of that, HPV E6 and E7 inactivate tumor suppressor genes p53 and retinoblastoma (Rb), respectively [10]. As a result of DNA damage, p53 expression increases, which causes G1 cell cycle arrest. As a result of this pause, DNA can be repaired, or apoptosis can be triggered in some cases [11]. Dephosphorylated Rb controls cell-cycle progression by inactivating growth-improving proteins such as E2F-1 and c-Myc transcription factors. Therefore, Rb functions to limit growth in normal cells, while its inactivation or absence in cancer cells promotes uncontrolled proliferation [12]. In addition to targeting tumor suppressors p53 and Rb, HPV can cause several cellular and molecular changes, such as genetic and epigenetic remodeling, during CC progression [13].

Several studies have demonstrated that microRNAs play a significant role in epigenetic remodeling [14]. These molecules are short single-stranded RNAs with about 22 nucleotides that negatively modulate post-transcriptional gene expression by targeting the 3′-untranslated mRNAs, resulting in destruction or translational suppression [15]. Moreover, they have been the subject of much research in cancer research, not only because they can function as oncogenes or tumor suppressor genes but also because they function within gene regulatory networks that control carcinogenesis through specific targets or signal pathways. They are involved in a variety of human malignancies because of their sine qua non roles [16].

Recently, several miRNAs have been abnormally expressed during CC development [17–19]. Additionally, various microRNAs exhibit distinct expression patterns between HR-HPV-positive CC cells and HPV-negative CC cells, as well as normal cervical tissues, throughout different stages of the disease. For instance, miR-18a showed increased expression following the suppression of HPV16 and 18 E6 or E7 oncoproteins during the transformation of HeLa cells [20]. Additionally, miR-34a played a role in distinguishing between low grade dysplasia and CC, particularly in patients with HPV16 and 18 infections [21]. This implies that HR-HPV-encoded proteins may directly influence miRNA expression within the host cell, which is essential to maintaining a transformed phenotype and subsequent progress to invasive carcinoma [22]. Moreover, the expression of mature miRNAs uniquely differs at different phases of CC development [23]. The findings suggest that CC may benefit from identifying numerous aberrantly expressed miRNAs induced by HR-HPV infection, which could be useful in early detection.

Two cancer-associated miRNAs, hsa-miR-194-5p and hsa-miR-195-5p, have been identified to be dysregulated in different malignancies, such as colon and prostate cancer, indicating they may act as potential diagnostic and prognostic biomarkers. Hsa-mir-194-5p expression is dysregulated in multiple malignancies and plays either as a tumor-suppressive or oncogenic factor in different cancers [24]. Hsa-mir-194-5p can also interact with essential signaling pathways in CC, such as the Wnt and Hippo signaling pathways [25]. p53 family members, directly affected by HPV oncogenes in CC development, are in an identical cluster with hsa-miR-194-5p in mammalian genomes [26]. Also, hsa-miR-195-5p is a miR-15/16 family member and may show some anti-cancer properties. However, this miRNA is downregulated in various cancers, such as melanoma, laryngeal, and colorectal cancer [27–29]. Furthermore, overexpression of the oncogene E2F transcription factor 3 (E2F3) promotes the development of different cancers and is related to the HPV *E6* and *E7* oncogenes [30]. Studies have shown that miR-194-5p and miR-195-5p inhibit Wnt, Hippo, and PI3K signaling pathways related to CC progression and development. These miRNAs have also been shown to affect E2F3 directly, making them ideal candidates for this study [28, 31].

With the aim of investigating the potential impact of hsa-miR-194-5p and hsa-miR-195-5p on CC progression, this study sought to evaluate the expression levels of these candidate microRNAs in Pap smear samples harboring HPV-16/18 infections and presenting normal cytology or severe dysplasia. The findings of this investigation shed light on the potential role of these microRNAs in cervical carcinogenesis, revealing new avenues for further research into the molecular mechanisms underpinning this devastating disease.

## Materials and method

### Sample procurement

In this study, 60 HPV-positive liquid-based cytology (LBC) samples (39 HPV16 and 21 HPV18) were collected from women with suspected cervical cancer from 2019 to 2021 at Afzalipour Hospital, Kerman, Iran. Ayre spatulas were used to obtain Pap smear samples from the surface of the cervix and its surrounding area. Of note, the patients received no prior pretreatment.

Inclusion criteria were as follows: i) all patients must have been infected with HPV16/18, which was confirmed by an HPV DNA test (INNO-LiPA HPV Genotyping Extra II kit; product number: 81534), and exclusion criteria were: i] individuals who had received radiation and chemotherapy before collecting specimens; ii) individuals suffering from acute organ failure; and iii] individuals who are cognitively and communicatively impaired.

Three pathologists evaluated all cases, and cytological outcomes were classified based on Bethesda classification [32].

Written consent was obtained from all individuals. All participants were also informed that all clinical data would be used only for scientific research. The study was approved by the Trabiat Modares University Ethics Committee under the following ID: IR.MODARES.REC.1400.306. This investigation was carried out based on the principles of the Declaration of Helsinki.

After applying exclusion criteria and microscopic examination, a total of 60 Pap smear samples were selected and classified into two groups: 1) the normal cytology group consists of 20 HPV16 and 10 HPV18; 2) the severe dysplasia (cervical intra-epithelial neoplasia 3 (CIN3)) group consists of 19 HPV16 samples and 11 HPV18 (each group, 30 samples). Then, samples were sent to the Stem Cell and Pathology Research Center in Kerman, Iran, for further analysis.

### RNA isolation

Total RNA was extracted from LBC samples using Trizol (15596026, Invitrogen, Carlsbad, CA, USA). RNA quality was verified by agarose gel electrophoresis, and its purity was confirmed by the relative absorbance ratio at A260/280 and A260/230 using Nanodrop 2000 (Thermo Scientific, USA); the acceptable ratio fell within the range of 1.8 to 2.0. Due to RNA instability, the obtained samples were instantly used for cDNA synthesis (Thermo Scientific). RNase-free conditions were maintained throughout the procedure.

### cDNA synthesis

Stem-loop primers were specifically designed for cDNA synthesis using miRNA sequence data from the miRbase database and the 44-nucleotide stem-loop structure developed by Chen et al. [33]. An extra six nucleotides were appended to the 5' end of the stem-loop sequence. These additional nucleotides were designed to have a sequence that is the reverse complement of the 3' end of the target microRNA. The physical properties of the resulting oligo sequences were then assessed using the OligoAnalyzer Tool (Table 1). PCR primers for hsa-miR-194-5p, hsa-miR-195-5p, and U6 snRNA were obtained from SinaClon Company, Tehran, Iran. Due to the steady expression of U6 small nuclear RNA in cervical tissues, it was chosen as the endogenous reference in this study [34].

**Table 1:** The sequence of used primers

Selected miRNAs	Sequence	
Hsa- miR-194-5P	RT	5’ GTCGTATGCAGTGCAGGGTCCGAGGTATTCGCACTGCATACGACTCCACA 3’
	F	5’ CACCATGTAACAGCAACTCC 3’
	R	5’ GTGCAGGGTCCGAGGT 3’
Hsa- miR-195-5P	RT	5’ GTCGTATGCAGTGCAGGGTCCGAGGTATTCGCACTGCATACGACGCCAAT 3’
	F	5’ AGAGAAGTAGCAGCACAGAAA 3’
	R	5’ GTGCAGGGTCCGAGGT 3’
U6 snRNA	RT	5’ GTGCAGGGTCCGAGGTTTGGACCATTTCTCGAT 3’
	F	5’ GGAACGATACAGAGAAGATTAGCA 3’
	R	5’ GTGCAGGGTCCGAGGT 3’

The manufacturer’s directions are to be followed (Thermo Scientific RevertAid First Strand cDNA Synthesis Kit): 1 μg of obtained RNA was added to 4 μl 5 × buffer, 1 μl reverse transcriptase enzyme, 1 μl of each miRNA cDNA synthesis specific primer, 2 μl dNTPs, and 10 µL RNase-free water to adjust the reaction volume to 20 µl. The combination was incubated in a thermal cycler at 44 °C for 60 min. To inactivate the reverse transcriptase enzyme, the mixture was kept for five minutes at 85 °C. Until needed, the cDNAs were stored at -20 °C.

### RT-qPCR

RT-qPCR was carried out by the Qiagene thermocycler (Rotor-Gene Q). The final volume was 20 μl, including 10 μl RealQ Plus 2 × Master Mix Green (Ampliqon, Denmark), 1 μl of each forward and reverse primer, 2 μl undiluted cDNA, and 6 μl RNase-free water to adjust the reaction volume to 20 µL. Rotor-Gene Q PCR cycling was used with the following steps: a 15-min denaturation at 95 °C, followed by 40 cycles of amplification at 95 °C for 15 s, 58 °C for 30 s, and 72 °C for 30 s. In order to assess primer efficiency, the Linear Regression of Efficiency (LRE) using the Windows-based LinRegPCR software was employed. The primer efficiency values obtained were 1.91 for miR-194 and 1.94 for miR-195.

Absolute quantification is not always required for miRNA expression analysis, as relative quantification can still provide valuable insights into differential expression patterns between sample groups. Moreover, absolute quantification often requires more starting material and may be more susceptible to variations in experimental conditions [35], leading to potential inaccuracies in the results. The 2^−ΔΔCT^ method was used in this study for analyzing RT-qPCR results. First, the average cycle threshold (Ct) values were calculated for the internal control and miRNAs. The Ct value is a cycle in which the fluorescence level reaches a certain amount. ∆Ct was the difference between the Ct values of each miRNA and the reference gene. 2^−∆Ct^, an expression of two studied miRNAs between our groups, was used [36].

### Hsa-miR-194-5p and Hsa-miR-195-5p functional enrichment

A DNA Intelligent Analysis DIANA-miRPath v3.0 program was used to characterize the functional properties of miRNAs (DIANA Lab, Thessaly, Greece) [37]. The Kyoto Encyclopedia of Genes and Genomes (KEGG) pathways, which the DIANA fully supports in the miRPath v3.0 database, were used to reach a better understanding of the function of dysregulated miRNAs and their putative shared targets [38]. The validated target pathways of hsa-miR-194-5p and hsa-miR-195-5p were identified using the algorithms of miRTarBase v7.0 and DIANA-microT-CDS. The DIANA-microT-CDS v5.0 algorithm is designed to recognize miRNA targets both in the 3′ untranslated regions and in coding sequences, based on complementary pairing with nucleotides in positions 1–9 at the miRNA 5′ end (based on complex physical models and/or machine learning approaches, microT-CDS can currently identify miRNA-gene interactions only in the 3′ UTR and CDS regions). The *P*-value and the false discovery rate < 0.05 were determined.

### Survival analysis using in-silico data

Using a Kaplan–Meier survival plot, the relationship between hsa-miR-194-5p and hsa-miR-195-5p expression and overall survival of CC was examined by in-silico data provided by Kaplan–Meier data base. This approach is applied when studying patient cohorts with varying time-to-event data, such as time until death or disease recurrence. The method calculates survival probabilities at different time points by considering observed events and censored data, producing Kaplan–Meier curves that illustrate survival trends [39]. The Kaplan–Meier Plotter database, as a broadly used online database, was used to evaluate the link between miRNA expressions and tumor prognosis. This database contains useful and reliable information from GEO, the European Genome-Phenome Archive (EGA), and TCGA. In order to reach the OS plot, we uploaded each miRNA into the Kaplan–Meier database. The overall survival (O) plot was generated by analyzing 307 CC samples available in the Kaplan–Meier database, using the miRpower tool within the pan-cancer functionality of the Kaplan–Meier plotter [40]. Log-rank *P*-values, hazard ratios, and 95% confidence intervals were calculated.

### Statistical analysis

Due to our study’s relatively small sample size, the Shapirowilk test was used to evaluate whether the data followed a normal distribution. In all groups, the data distribution had a non-normal distribution. Hence, the Mann–Whitney test was used to examine differences in the mean between miRNAs expression (hsa-miR-194-5p and hsa-miR-195-5p) and ∆Ct in CC samples, HPV-positive with normal and dysplasia. GraphPad Prism version 9 was used for statistical analysis. Receiver operating characteristic (ROC) curves were generated to assess the sensitivities and specificities of individual miRNAs as well as their combinations.The area under the ROC curves (AU) was calculated for the prediction of the cut-off values of the markers. A significant level was defined as a *P*-value of less than 0.05 for all tests.

## Results

### The expression levels of hsa-miR-194-5p and hsa-miR-195-5p decreased in CC patients

As an effective approach, integrative computational bioinformatics procedures were used to detect nominated miRNAs using large-scale expression profiling data and low-throughput experimental verification. Moreover, according to the literature review, the proposed possible targets, and the earlier scientific discoveries about miRNAs contributing to CC progression, we selected hsa-miR-194-5p and hsa-miR-195-5p as candidate miRNAs.

**Fig. 1:**
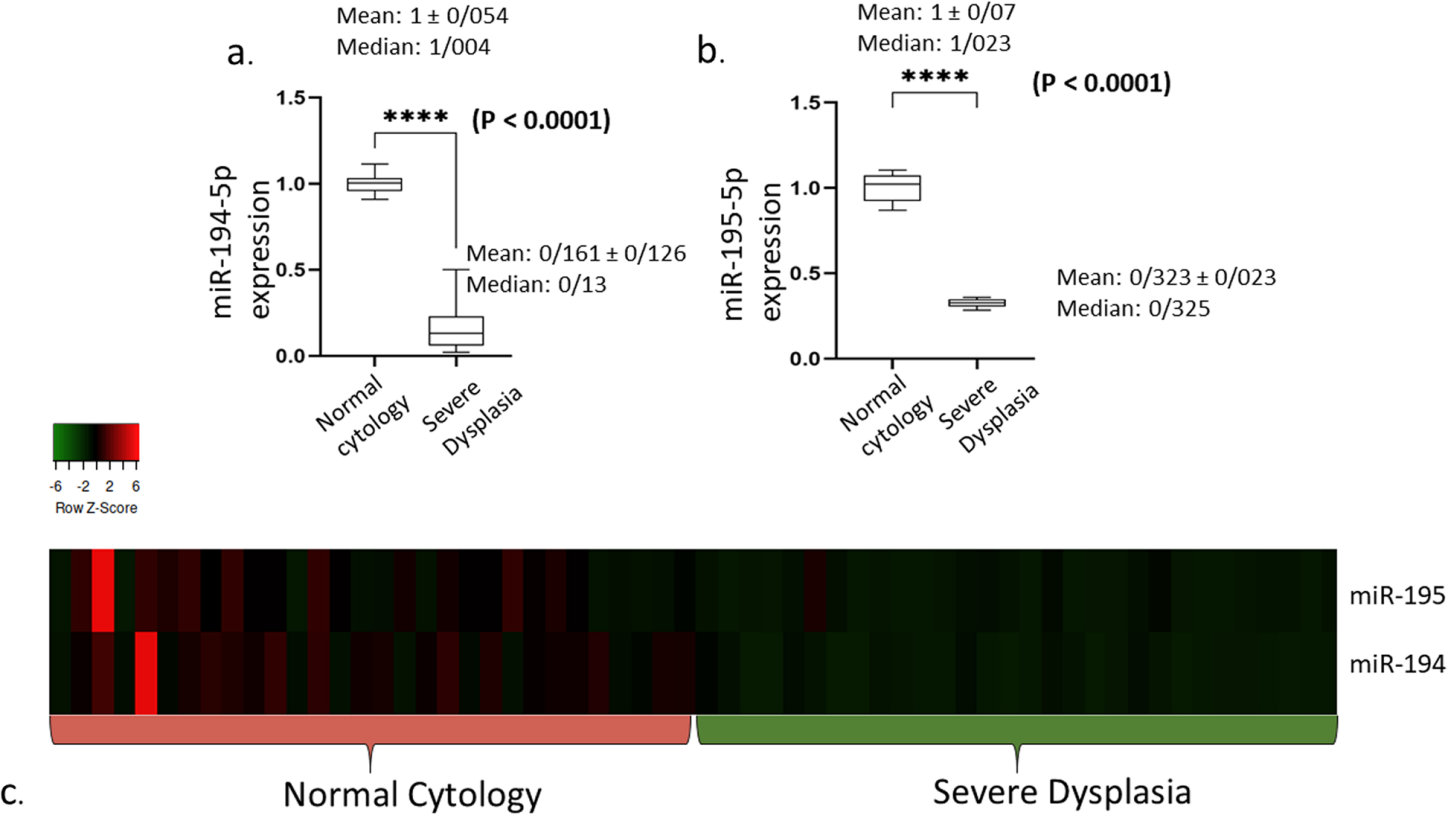
candidate miRNAs expression profile in the HPV-Positive Pap smear samples of patients with normal cytology and severe dysplasia. **a** and **b** expression analysis indicated a significant down-regulation of hsa-miR-194-5p (fold change: -2.628, *P* < 0.0001) and hsa-miR-195-5p (fold change: -1.584, *P* < 0.0001) in the HPV-Positive patients with severe dysplasia compared to normal cytology controls (median, mean, and standard deviation values displayed above each box plot). **c** HPV-positive individuals with severe dysplasia and normal cytology were distinguished using unsupervised hierarchical cluster analysis employing differentially expressed miRNAs. Red indicates miRNAs with relatively high expression, while miRNAs with low relative expression are shown in green on the heatmap (Euclidian distance, complete linkage). Heatmapper (http://www.heatmapper.ca) was used to make the heatmap

According to the literature review, mature miR-194 and miR-195 were expressed ubiquitously in all samples. Thus, their expression was normalized using U6 snRNA. RT-qPCR was used to examine the expression status of these miRNAs between HPV-positive Pap smear samples with severe dysplasia and cytologically normal HPV-positive samples as controls. Hsa-miR-194-5p (fold change: -2.628, *P* < 0.0001) and hsa-mir-195-5p (fold change: -1.584, *P* < 0.0001) were significantly down-regulated in samples with severe dysplasia compared with controls (Fig. 1a and b). Unsupervised hierarchical cluster analysis revealed that the pattern of significantly lower expression of selected miRNAs in Pap smear samples could be used to distinguish progressive cervical lesions from HPV-positive cytologically normal samples (Fig. 1c). Comparisons between patients’ demographic and clinical data are shown in Table 2.

**Table 2:** Comparison of patient’s general information using the Chi-Square experiment

		Cellular changes		X^2^	*P*-Value
		Normal	Severe		
Age	≤ 45	11 (36.7%)	14 (46.7%)	0.617	0.432
	> 45	19 (63.3%)	16 (53.3%)		
Active Smoker	No	20 (66.7%)	23 (76.7%)	0.738	0.39
	Yes	10 (33.3%)	7 (23.3%)		
Family History of CC	No	22 (73.3%)	22 (73.3%)	0.000	1.000
	Yes	8 (26.7%)	8 (26.7%)		
Marital Status	Single	1 (3.3%)	4 (13.3%)	1.963	0.161
	Married	29 (96.7%)	26 (86.7%)		

### Hsa-miR-194-5p and hsa-miR-195-5p target prediction and linked pathways

**Fig. 2:**
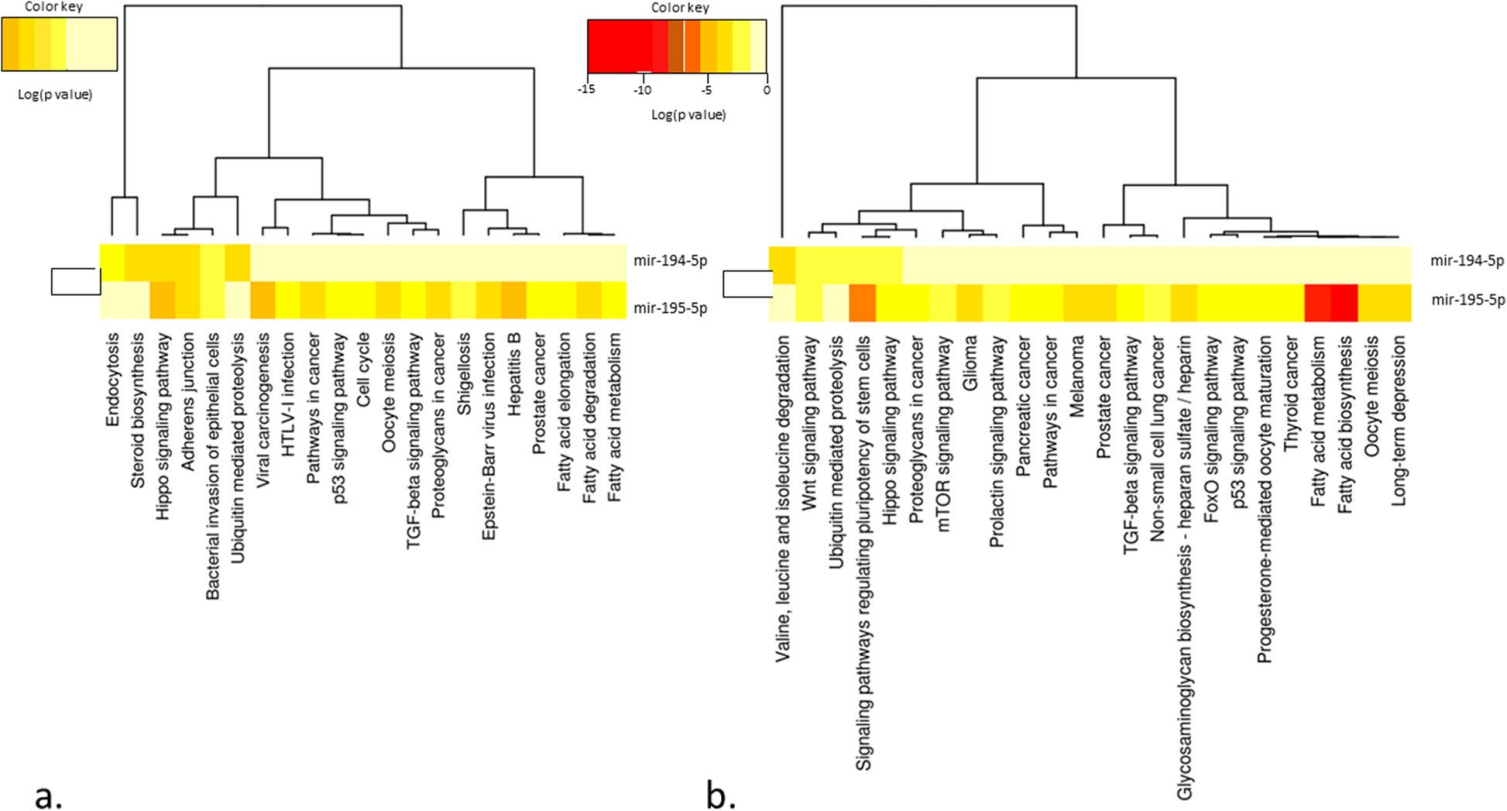
Heatmap representing differently expressed miRNAs against substantially enriched functional networks. **a** Hierarchical clustering results for each miRNAs and their relevant pathways are shown. Hippo signaling pathway and adherens junction pathway were overlapped pathways between candidate miRNAs. The figure depicted is based on the online output of Diana miRpath V.2 using the algorithm of miRTarBase v7.0. **b** Predicted pathways heat map DIANA-microT-CDS (v5.0) algorithm. Based on this algorithm, the wnt signaling pathway and signaling pathways regulating the pluripotency of stem cells were common among candidate miRNAs. Lower *P* values (more significant) and higher interplay of each miRNA with a particular molecular pathway are indicated by reddish color combinations in the heatmap

**Fig. 3:**
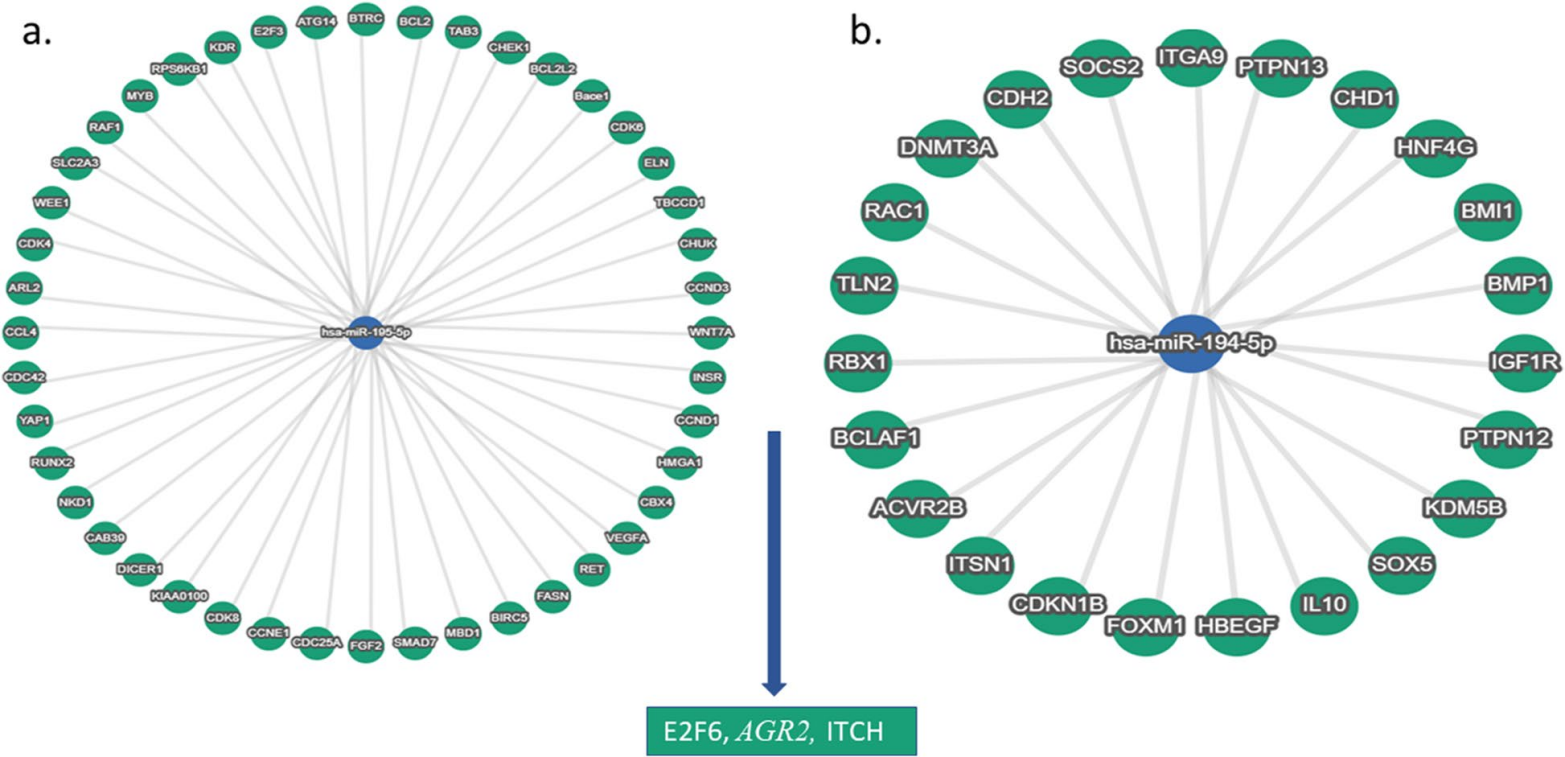
miRNAs validated and predicted target genes. **a** the strong interplay of hsa-miR-195-5p with its validated target genes provided by miRTargetLink 2.0 Human. **b** The same network depicted for hsa-miR-194-5p. **c** After predicted interaction comparison, E2F6, AGR2, and ITCH genes have remained. hsa-miR-194-5p and hsa-miR-195-5p did not show any strong and weak interaction in this network (weak and predicted interactions are not depicted here)

In the current study, we used DIANA-miRPath v3.0 (http://www.microrna.gr/miRPathv3), an online software package designed to evaluate miRNA regulatory roles and identify controlled pathways. More than 600,000 miRNA targets have been experimentally supported in DIANA-TarBase v7.0. In order to compare the results of DIANA-TarBase v7.0 with high-quality in silico predicted targets, we additionally employ DIANA-microT-CDS [38]. We also used DIANA-miRPath v. 3.0 software to undertake a computational target prediction study and a pathway analysis. We illustrated the shared pathways between hsa-miR-194-5p and hsa-miR-195-5p using the programs of miRTarBase v. 7.0 and DIANA-microT-CDS (v5.0). The paths are clustered according to significance levels, and the findings are presented as heatmaps in Fig. 2a and b. MirTargetLink 2.0 [41] was used to map the anticipated mRNA targets of the verified miRNAs. There were no valid overlapped gene targets between the miRNAs (Fig. 3a and b).

### Hsa-miR-194 correlates with overall poor survival in computational analysis of cervical cancer patients

Since our data supports that hsa-miR-194 and hsa-miR-195 expression is downregulated with advanced stages of cervical dysplasia and they may target some critical genes and signaling pathways, we hypothesized whether these miRNA expression levels affect CC patients’ survival or not. We assessed the predictive capacity of specific miRNAs by leveraging the Kaplan–Meier plotter database, which draws on comprehensive datasets from previous patient studies.Survival curves were plotted for CC individuals provided by Kaplan–Meier data base [40]. Interestingly, in-silico data showed that OS was low in individuals with lower expression of hsa-miR-194. The data also showed that the median survival time of CC patients with low miR-194 expression was 27.9 months (*p* < 0.01), which was significantly shorter than those with higher miR-194 expression, 48.43 months (*p* < 0.01, false discovery rate (FDR) < 0.3, no analysis restriction was applied). According to previous studies, the low expression of miR-194 in different malignancies, such as colorectal and breast cancer, is inversely correlated with q.

**Fig. 4:**
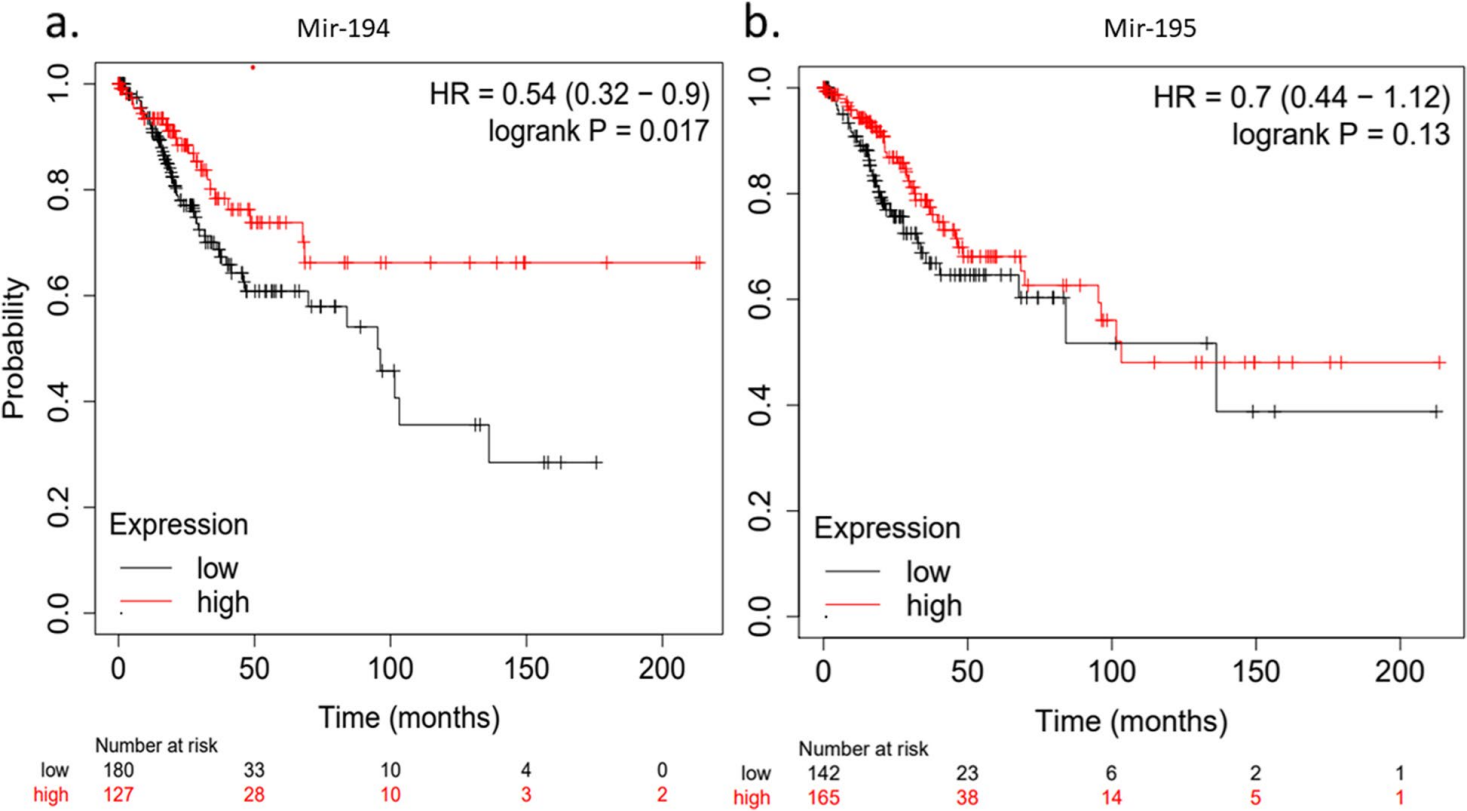
Kaplan–Meier survival curve indicates the prognostic value of the hsa-miR-194 in CC using the Pan-cancer analysis in mirPower, which is a part of the Kaplan–Meier plotter database to analyze the prognostic value of the desired miRNAs in a different type of cancer over time, *p*-value > 0.05 (**a**) Downregulation of the hsa-miR-194 is related to the poor overall survival of CC. High expression of hsa-miR-194 was identified in 127 patients, while 180 patients showed low expression for this miRNA. **b** hsa-miR-195-5p was not a reliable prognostic biomarker in CC (hazard ratio; 95% and confidence interval showed in parentheses)

The relationship between the downregulation of the hsa-miR-195 and overall poor CC survival was insignificant. The duration of the follow-up threshold was set at 200 months. The graphs showed that as the CC progresses, the OS diminishes (Fig. 4). It should be considered that the Kaplan–Meier plotter does not distinguish between the -5p and 3p forms of the microRNAs.

### Diagnostic value of miR-194-5p and miR-195-5p in CC

**Fig. 5:**
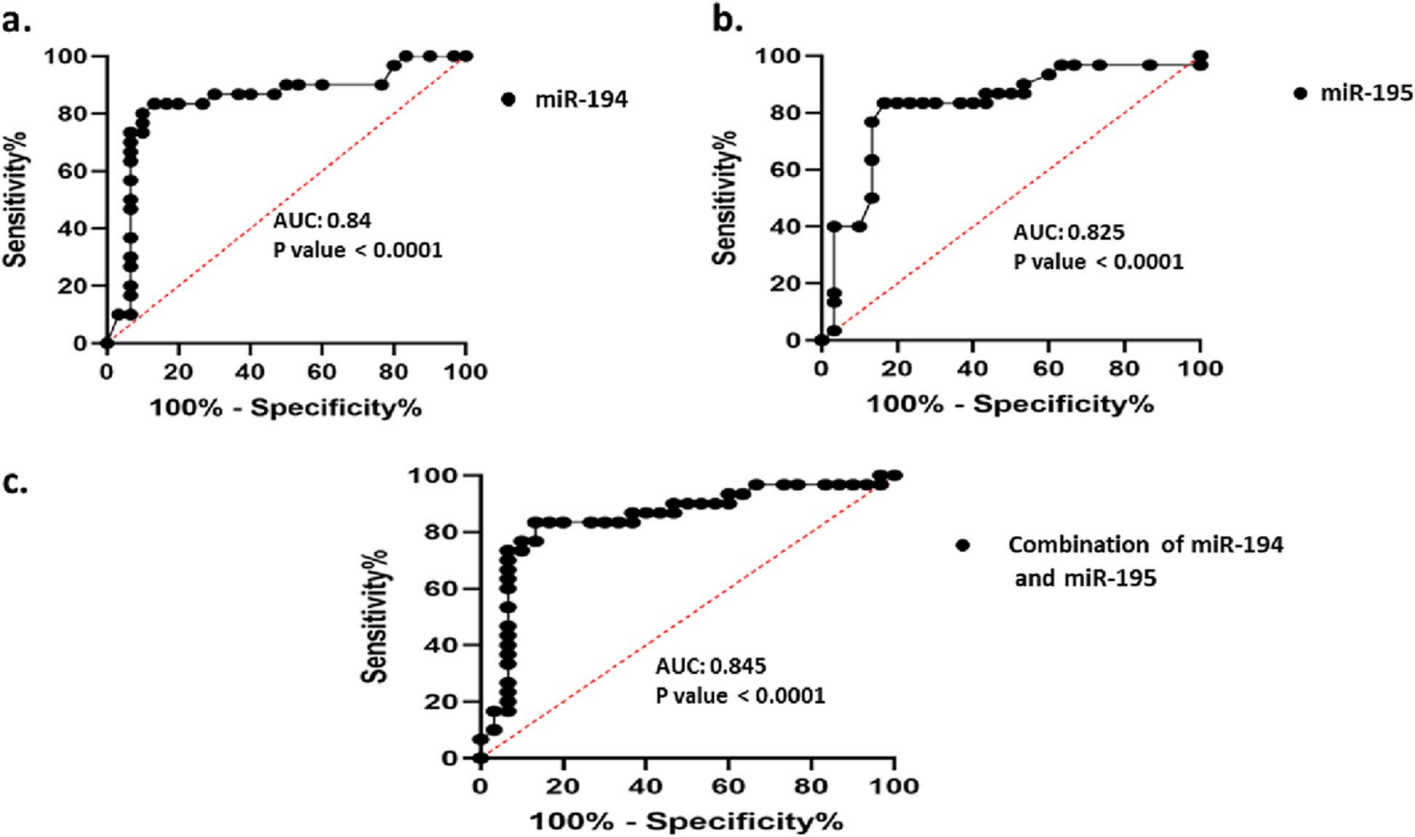
ROC curves were constructed to indicate miR-194 (**a**) and miR-195 (**b**) AUCs. **a** At the cut-off value of 0.41 for hsa-miR-194-5p, the sensitivity was 80%, and the specificity was 90% with an AUC of 0.84 (95% CI = 0.727–0.935). **b** At the cut-off value of 0.695 for hsa-miR-195-5p, the sensitivity, and specificity were 83.33 (95% CI = 0.712–0.937). **c** The combination of miR-194 and miR-195. At the cut-off value of 0.432, the sensitivity for combined form was 83.33% and the specificity was 86.67%, with an AUC of 0.845 (95% CI = 0.736–0.954)

To evaluate the diagnostic value of a miRNA expression signature in cervical dysplasia development, ROC curve analysis was done. According to the ROC curves, results (based on the sensitivity and specificity of miRNA expression) indicated that the AUCs were 0.840 (*P* < 0.0001) and 0.825 (*P* < 0.0001) for miR-194-5p and miR-195-5p, respectively (Fig. 5a and b). At the cut-off value of 0.41, the optimal sensitivity and specificity for miR-194-5p were 80% and 90% (95% confidence interval (CI) = 0.727–0.953), respectively. At the cut-off value of 0.695, both sensitivity and specificity for miR-195-5p were 83.33% (95% CI = 0.712–0.937). For optimal diagnostic accuracy, the combination of miR-194-5p and miR-195-5p was employed, resulting in a modest improvment in diagnostic accuracy. The combined miRNA approach yielded an AUC value of 0.845 (*p* < 0.0001). At a specific cut-off value of 0.432, the miRNA combination exhibited a sensitivity of 83.33% and specificity of 86.67% (Fig. 5c).

## Discussion

CC is a genetic and pathologic condition in which dysregulation of miRNAs may play a key role in different aspects of cancer hallmarks [42]. To diagnose precancerous cervical lesions, many techniques (e.g., Pap smear, colposcopy, and biopsy) are now widely used [43, 44]. However, the limited specificity and sensitivity of these approaches and their invasive nature are key drawbacks [43, 45]. Therefore, this malignancy remains one of the world’s most significant health issues because most HR HPV infections present with no symptoms after one to two years [46].

The dysregulation of miRNAs has been firmly established in various stages of under-developed CC patients, indicating their critical involvement in the development of this disease [47]. According to Wilting et al., chromosomal instability alters miRNA expression at different stages of cancer growth [48]. Studies suggest a correlation between viral oncoproteins and the dysregulation of miRNAs in various stages of cervical dysplasia, which may contribute to the development and progression of CC [49]. HPV-induced cell transformation requires the viral oncoproteins E6 and E7. E6 induces the proteolytic degradation of the p53 tumor suppressor protein, which plays a vital role in the transcription of numerous coding and non-coding genes. Also, E7 inhibits the retinoblastoma tumor suppressor from releasing E2F from the pRb-E2F complex; thus, HPV E6 and E7 can modulate miRNA expressions [8].

Since miRNAs are more stable and accessible to detect than mRNA, they may be a promising candidate for cancer diagnosis [50]. A computational bioinformatics approach has been used to identify miRNAs that may contribute to CC development. As a logical strategy, candidate miRNAs (hsa-miR-194-5p and hsa-miR-195-5p) were chosen using preliminary detection of candidate miRNAs derived from large-scale expression profile data and low-throughput experimental verification for the selected miRNAs. In addition, hsa-miR-194-5p and hsa-miR-195-5p target members of the E2F transcription factor family and p53, a tumor suppressor protein [51, 52]. In addition, early research indicates that hsa-miRNA-194-5p and hsa-miRNA-195-5p play an instrumental role in many signaling pathways commonly dysregulated in different stages of CC development, including Wnt, PI3K, and Hippo signaling pathways [25, 53]. Most cancer and miRNA investigations are conducted on cell lines and tissue specimens. While these studies have considerably helped raise the scientific findings on cancer, using tissue for cancer diagnosis is an invasive procedure. To our knowledge, there is no published report on Pap smear samples using RT-qPCR to analyze hsa-miR-194-5p and hsa-miR-195-5p expression in HPV-positive patients with normal cytology and pre-malignant lesions.

Hsa-miR-194-5p and hsa-miR-195-5p may target some critical genes related to HPV infection and cancer progression, according to the bioinformatics predictions. Using DIANA-miRPath v3.0 and miRTargetLink 2.0, we suggested *E2F6, anterior gradient 2 (AGR2),* and *ITCH* (also known as atrophin1-interacting protein 4; AIP4) as putative targets for hsa-miR-194-5p and hsa-miR-195-5p. E2F6 is a member of polycomb complexes, attaches to repressed chromatin, and is essential for cell fate and cell proliferation [54]. In addition, McLaughlin-Drubin et al. provided evidence that HPV E7 interacts with and functionally deregulates the *E2F* family member *E2F6* [55]. While other research indicates that hsa-miR-195-5p can interact with various genes (such as *NEK2*, *LOXL2*, *YAP1*, and *PFKFB4*) in CC, the decrease in its expression was consistent across all investigations and among us [52]. Furthermore, down-regulation of hsa-miR-194-5p expression was closely associated with E2F3 up-regulation and node metastasis in bladder cancer [31].

Abnormal hyperplasia of the cervical squamocolumnar (SC) junction, vulnerable to HR-HPV, strongly correlates with CC. The Liu et al. study revealed that the SC junction marker *AGR2* is overexpressed during CC progression [56]. Interestingly, Li et al. showed that *AGR2* could be negatively regulated by hsa-miR-194-5p in colorectal cancer [57].

ITCH belongs to the Nedd4-like family of E3 ubiquitin ligases and plays a crucial role in tumorigenesis [58]. Zhou et al. identified a gradually increased expression of *ITCH* during CC tumorigenesis, which negatively correlated with large tumor suppressor 1 (LATS1, a critical tumor suppressor in CC) [59]. Yes-associated protein (YAP), a key downstream effector of the Hippo signaling pathway, is phosphorylated by activated LATS1, which causes this protein to get degraded [60]. Furthermore, based on miRTarBase v7.0 and microT-CDS v5.0 algorithms, the Hippo signaling pathway and the WNT signaling pathway overlapped between our candidate miRNAs. According to He et al., YAP expression significantly increased as CC progressed. They also confirmed that YAP overexpression is directly related to the HPV16 E6 oncoprotein [61], indicating that the Hippo signaling pathway could be crucial in CC development. YAP may also be directly targeted by hsa-miR-194-5p and hsa-miR-195-5p and negatively regulated by them [62, 63].

Mutated or deregulated Wnt signaling components are extensively characterized in CC; HPV E6 oncoprotein can also stimulate the Wnt signaling pathway using a variety of mechanisms, highlighting the great importance of the Wnt signaling pathway in CC development [64]. Previous studies demonstrated that hsa-miR-194-5p and hsa-miR-195-5p could deregulate the Wnt/β-catenin pathway in various cancers [65, 66]. Overall, we believe that hsa-miR-194-5p and hsa-miR-195-5p modulate *E2F6*, *AGR2*, and *ITCH* to perform their activities in HPV-positive under-development CC patients. Moreover, the hippo and Wnt signaling pathways are common target pathways among our candidate miRNAs. However, more functional experiments modulating hsa-miR-194-5p and hsa-miR-195-5p are needed to confirm.

A ROC curve analysis was used to evaluate the diagnostic accuracy of the candidate miRNAs. These results are consistent with earlier research showing that miR-194-5p and miR-195-5p expression is downregulated in various malignancies [67], suggesting them as probable diagnostic markers for CC. Furthermore, it was demonstrated that the diagnostic accuracy achieved by combining miRNAs (hsa-miR-194 and hsa-miR-195) was slightly superior to that of individual miRNAs. According to data from the Kaplan–Meier plotter online database, low expression of hsa-miR-194 is associated with poor survival in CC patients. Several malignancies, including colorectal and ovarian cancer, have a poor prognosis related to hsa-miR-194 reduction [68, 69]. It is important to note that the Kaplan–Meier plotter analysis does not distinguish between the -5p and -3p forms of the microRNAs. In a study conducted by Azimi et al., it was observed the level of miR-195-3p (2.82, *P* = 0.01) in Pap smear samples was significantly higher in patients with high-grade squamous intraepithelial lesions (HSIL) compared to those with low-grade squamous intraepithelial lesions (LSIL) [70]. Considering these facts, it makes sense that the Kaplan–Meier plotter did not show a significant association between miR-195-5p reduction and overall poor survival in CC.

According to the literature, hsa-miR-194-5p and hsa-miR-195-5p are frequently dysregulated in numerous malignancies and can operate as tumor suppressors [71, 72]. It was discovered in the study of Song et al. that hsa-miR-195-5p functions as a tumor suppressor in CC, and its expression in CC tissues and cell lines decreased compared with control groups [73]. Additionally, Yang et al.’s investigation revealed that miR-195 expression in CC tissues is markedly downregulated in the early stages of CC [74]. These are entirely in line with our findings in Pap smear samples. There was also a study that examined the relationship between hsa-miR-195-5p, *MMP14* (matrix metalloproteinases 14), and *HDGF* (hepatocellular growth factor) [73, 75]. Interestingly, MMP14 (a significant contributor to the invasion and migration of CC cells) and HDGF (an overexpressed transcription factor in CC) can insert their effects through the Rb-E2f pathway [73]. The downregulation of hsa-miR-195-5p results in the overexpression of *MMP14* and *HDGF*, which act as tumor promoters and are both targeted by hsa-miR-195-5p. As the expression of ADP-ribosylation factor-like 2 (*ARL2*) decreases, hsa-miR-195-5p limits CC cell malignancy. [76]. In another scenario, the plasmacytoma variant translocation 1 gene, considered an oncogenic long noncoding RNA, is induced by the HPV16 E7 gene, significantly reducing the number of hsa-miR-195-5p expressions [77]. Therefore, hsa-miR-195-5p inhibits CC formation by targeting various downstream proteins and can act as a tumor-suppressor agent. In our study, hsa-miR-195-5p expression was significantly decreased in HPV-positive with severe dysplasia LBCs compared to HPV-positive LBCs with normal cytology.

Hsa-miR-194-5p is downregulated in gliomas and targets *Bmi1* to decrease epithelial-to-mesenchymal transition. This miRNA is also downregulated in colorectal cancer (CRC) and targets *KLK10* to inhibit its proliferation, verifying its tumor-suppressive roles [78, 79]. In prostate cancer, on the other hand, hsa-miR-194-5p is increased and downregulates *SOCS2* to promote cancer development [80]. Zhang et al. discovered that the SLC16A1-AS1 long non-coding RNA sponges hsa-miR-194-5p, and the downregulation of this long non-coding RNA in cervical cancer leads to hsa-miR-194-5p overexpression, which improves cancer cell development by *SOCS2* downregulation [81]. These findings contradicted our findings, showing a significant drop in hsa-miR-194-5p expression in HPV-positive cytologically abnormal LBCs compared to HPV-positive LBCs with normal cytology. Hsa-miR-194-5p seems to be a target for P53 that modulates some p53 biological actions, including the induction of growth arrest and apoptosis [82]. The HPV E6 protein forms a compound with the cellular proteins E6-AP and p53, allowing p53 to be degraded more quickly through the ubiquitin-dependent proteolytic pathway [83]. Therefore, it is tempting to believe that hsa-miR-194-5p has tumor-suppressive properties in HPV-positive CC. However, few studies show how hsa-miR-194-5p plays during CC development. Still, one scenario based on Wang et al.’s study on prostate cancer suggests that downregulation of hsa-miR-194-5p is linked to overexpression of the oncogene E2F transcription factor 3 (E2F3), which can improve cancer progression [31, 84]. Because the E2F transcription factor family is targeted by viral oncoprotein E7, we hypothesized that hsa-miR-194-5p could also play a tumor-suppressive role in CC.

## Conclusion

In essence, evaluating the expression level of hsa-miR-194-5p and hsa-miR-195-5p in HPV-positive cytologically abnormal LBCs compared to HPV-positive LBCs with normal cytology in women from the south-east of Iran was a fresh idea in this investigation. There was a significant reduction of the selected miRNAs in severe dysplasia cytology HPV-positive samples compared to HPV-positive samples with normal cytology, suggesting their potential tumor-suppressive role in CC. However, we need more research to determine how HPV-related progression in CC works. Future research could confirm their tumor-suppressive properties by modulating hsa-miR-194-5p and hsa-miR-195-5p in CC cells.

## Data Availability

The data can be made available upon reasonable request from the Corresponding author.

## Abbreviations

CC: Cervical cancer
HPV: Human papillomavirus
HR-HPV: High-risk HPV
Rb: Rretinoblastoma
LBC: Liquid based cytology
CIN3: Cervical intra-epithelial neoplasia 3
Ct: Cycle threshold
EGA: European Genome-phenome Archive
ROC: Receiver operating characteristic
AUC: The area under the ROC curves
FDR: False discovery rate
AGR2: Anterior gradient 2
AIP4: Atrophin1-interacting protein 4
SC: Squamocolumnar
LATS1: Large tumor suppressor 1
HSIL: High-grade squamous intraepithelial lesions
LSIL: Low-grade squamous intraepithelial lesions
MMP14: Matrix metalloproteinases 14
HGDF: Hepatocellular growth factor
ARL2: ADP-ribosylation factor-like 2
CRC: Colorectal cancer
